# Complement-targeted therapies for transplant-associated thrombotic microangiopathy: recent advances

**DOI:** 10.3389/fphar.2026.1835245

**Published:** 2026-06-15

**Authors:** Taichiro Tokura, Sayuri Motomura

**Affiliations:** 1 Department of Hematology, Tokyo Metropolitan Tama-Hokubu Medical Center, Tokyo, Japan; 2 Department of Hematology, Nippon Medical School, Tokyo, Japan

**Keywords:** C5 inhibition, complement inhibition, complement pathway, eculizumab, MASP-2 inhibition, narsoplimab, ravulizumab, transplant-associated thrombotic microangiopathy (TA-TMA)

## Abstract

Transplant-associated thrombotic microangiopathy (TA-TMA) is a life-threatening complication of hematopoietic stem cell transplantation in which systemic complement activation induces endothelial injury, leading to microangiopathic hemolytic anemia, thrombocytopenia, and organ dysfunction. TA-TMA often progresses rapidly and has historically been associated with high mortality. Over the last decade, increasing recognition of complement activation as a central driver of TA-TMA has shifted management from largely empirical supportive care toward mechanism-based therapy. This review summarizes recent advances in complement-targeted therapies for TA-TMA and outlines key aspects of pathophysiology, risk factors, and monitoring that inform therapeutic decision-making. Earlier non–complement-directed therapies, including calcineurin inhibitor modification, plasma exchange, defibrotide, and rituximab, showed limited and inconsistent benefit. In contrast, complement-targeted therapies have advanced the treatment of TA-TMA. Prospective and large cohort data support the clinical activity of eculizumab (C5 inhibitor), with meaningful improvements in survival and organ recovery. Ravulizumab (long-acting C5 inhibitor) has shown encouraging phase 3 results, and narsoplimab (MASP-2 inhibitor), which became the first approved treatment for TA-TMA, has demonstrated promising outcomes, including activity in some patients previously exposed to C5 inhibition. In addition, newer agents targeting C5, C3, or factor B are expanding the therapeutic horizon for TA-TMA, although efficacy data are still limited for some of these therapies. Overall, complement-targeted therapies represent a therapeutic advance in TA-TMA, and ongoing prospective studies will be crucial to define optimal agent selection, sequencing, and integration into clinical practice.

## 1 Introduction

Transplant-associated thrombotic microangiopathy (TA-TMA) is a life-threatening complication of hematopoietic stem cell transplantation (HSCT). Since the 2000s, it has been recognized that a highly fatal TMA resembling thrombotic thrombocytopenic purpura (TTP) and hemolytic uremic syndrome (HUS)—characterized primarily by microangiopathic hemolytic anemia (MAHA), thrombocytopenia, and renal failure—may develop after HSCT. During the 2010s, various therapeutic approaches were attempted, but prognosis remained poor. Over the last decade, the pathophysiology has been elucidated, with therapeutic advances. Complement-targeted therapies tailored to the pathophysiology have improved outcomes. Several groups, including the Blood and Marrow Transplant Clinical Trials Network (BMT-CTN) (Ho et al., 2005) and the International Working Group (IWG) (Ruutu et al., 2007), proposed diagnostic criteria, culminating in the 2022 harmonized criteria (Schoettler et al., 2023). In 2025, consensus response criteria were also proposed (Schoettler et al., 2025a). These advances highlight progress in TA-TMA research, and complement-targeted trials are ongoing.

Recent studies using contemporary diagnostic criteria and prospective screening suggest that the incidence of TA-TMA may be higher than previously appreciated. In adults, the MIDAS Consortium study, the first prospective study evaluating TA-TMA in adults using the 2022 harmonized criteria, reported a 100-day incidence of severe TA-TMA of 21.8% (Vasu et al., 2025). In contrast, an external validation study using the same criteria reported an incidence of 6.2% after allogeneic HSCT (Acosta-Medina et al., 2025). In children, a pragmatic prospective multicenter study reported an incidence of 16% (Dandoy et al., 2021). Given its high mortality, TA-TMA remains a substantial threat. This review summarizes recent progress in complement-targeted therapies that have improved outcomes in TA-TMA.

## 2 Pathophysiology and risk factors

TA-TMA is a complement-mediated disorder in which systemic complement activation induces endothelial injury, vasculopathy, and microvascular thrombosis (Jodele and Sabulski, 2023). Microthrombi in the microcirculation cause MAHA, thrombocytopenia, and multiple organ dysfunction syndrome. Once organ injury develops, interferons and other pro-inflammatory cytokines are released, further amplifying complement activation and worsening disease (Jodele and Sabulski, 2023).

**FIGURE 1:**
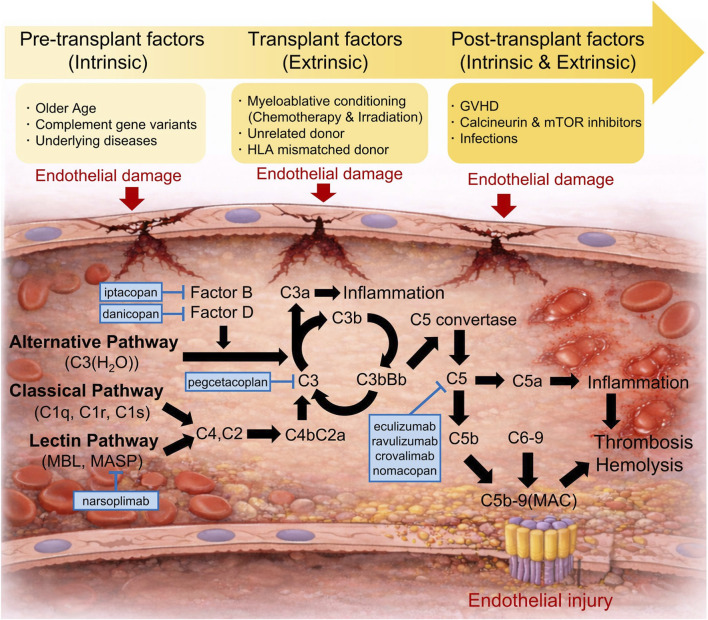
Pathophysiology and risk factors of transplant-associated thrombotic microangiopathy (TA-TMA).

Complement activation in TA-TMA proceeds through the alternative, classical, and lectin pathways (Figure 1). Key effectors are C3a, C3b, C5a, and C5b-9 (Mahmoudjafari et al., 2023). C3a and C5a are anaphylatoxins that amplify inflammation and promote thrombosis, leukocyte recruitment, and endothelial activation. During inflammation, activated neutrophils release granular proteins and form neutrophil extracellular traps (NETs), which can contribute to endothelial injury and further amplify complement activation (Ibrahimova et al., 2026; Yuen et al., 2016). C3b promotes phagocytosis and contributes to C5 convertase formation. C5b-9 is the terminal complement complex that forms the membrane attack complex (MAC), creates transmembrane pores, and promotes endothelial injury and microvascular thrombosis (Jodele and Sabulski, 2023).

In this context, systemic complement activation is central to TA-TMA pathophysiology. However, it can be initiated or intensified by multiple factors. Here, we classify these factors as (i) pre-transplant, (ii) transplant, and (iii) post-transplant.

Pre-transplant factors are intrinsic factors present before transplantation. Several studies reported greater susceptibility in females (Van Benschoten et al., 2022; Epperla et al., 2020). Older age may likewise increase risk (Van Benschoten et al., 2022). Genetic predisposition related to complement-gene variants has been reported: the presence of at least one variant is associated with TA-TMA susceptibility, and increased numbers of variants contribute to higher mortality (Jodele et al., 2016). Underlying diseases such as primary immunodeficiency and severe aplastic anemia may predispose to TA-TMA (Dandoy et al., 2021; Higham et al., 2021).

Transplant factors are extrinsic factors, including conditioning chemotherapy, radiation, and donor-related characteristics. Myeloablative conditioning causes greater endothelial damage than reduced-intensity conditioning and has been linked to increased TA-TMA risk (Van Benschoten et al., 2022). Total body irradiation (TBI) and fludarabine have also been implicated in endothelial damage (Kafa and Hoell, 2024; Liu et al., 2024). Prior HSCT and tandem autologous HSCT for neuroblastoma are associated with a higher incidence, plausibly reflecting cumulative endothelial damage from repeated chemo-radiotherapy (Van Benschoten et al., 2022; Dandoy et al., 2021). Unrelated and HLA-mismatched donors have been identified as additional risk factors (Van Benschoten et al., 2022; Epperla et al., 2020). Because ABO antigens are expressed on endothelial cells, donor–recipient ABO incompatibility may exacerbate endothelial injury, although contemporary data directly linking ABO mismatch to TA-TMA are limited.

Post-transplant factors comprise heterogeneous risks after transplantation, including medications and transplant-related complications, and encompass both intrinsic and extrinsic components. Calcineurin inhibitors (CNIs) such as tacrolimus and cyclosporine, as well as the mTOR inhibitor sirolimus, have been implicated in TA-TMA (Van Benschoten et al., 2022; Heybeli et al., 2020). Acute and chronic graft-versus-host disease (GVHD) also contribute to risk (Heybeli et al., 2020), with high-grade acute GVHD showing a particularly strong association in some cohorts (Van Benschoten et al., 2022). Viral and other infections are likewise important contributors (Van Benschoten et al., 2022). In particular, BK virus has been prospectively identified as a risk factor and warrants heightened clinical awareness (Laskin et al., 2020). At this stage, intrinsic and extrinsic factors that can promote complement activation, such as drugs, GVHD, and infections, are closely intertwined in both the onset and deterioration of TA-TMA.

Importantly, this framework differs somewhat from the recently proposed three-hit hypothesis (Dvorak et al., 2019). A major distinction is that a stepwise sequence of three hits may imply that all three components are required for TA-TMA to occur, whereas TA-TMA can develop even when some are absent. In other words, not all three “hits” are necessarily required for TA-TMA to develop. For example, TA-TMA can occur after autologous HSCT, where GVHD and immunosuppressant exposure—considered the “third hit”—are absent, implying that endothelial damage alone can be sufficient in selected settings. Moreover, factors often categorized as the third hit, such as GVHD and infections, may act as both triggers and exacerbating drivers once TA-TMA has developed. In our experience treating TA-TMA after autologous HSCT with C5 inhibitors, disease control appeared more straightforward than after allogeneic HSCT. This may be because allogeneic HSCT is more frequently complicated by GVHD, diverse infections, and polypharmacy than autologous HSCT, all of which can sustain complement activation and make suppression more difficult. Therefore, we regard post-transplant factors as important exacerbating drivers and possible initiating factors. This view has therapeutic implications: after TA-TMA onset, complement inhibition should be accompanied by measures to reduce post-transplant factors as much as possible to prevent further complement activation.

## 3 Assessing biomarkers of complement activation in the diagnosis of TA-TMA

Because TA-TMA is a complement-mediated disorder, its diagnosis should be based on multiple findings rather than any single feature in isolation (Schoettler et al., 2023). In current practice, TA-TMA is diagnosed based on a combination of findings, including MAHA, thrombocytopenia, organ injury, and, when available, biomarkers reflecting complement activation. Because these abnormalities may emerge at different time points during disease evolution, prospective TA-TMA screening is critical for timely diagnosis and interpretation of dynamic changes in hematologic, renal, and complement-related parameters before overt organ dysfunction develops.

Among findings suggestive of MAHA, schistocytes are a classic feature and were observed in 91.9% of one adult cohort (Heybeli et al., 2020). However, schistocytes are not always an early or reliable marker of TA-TMA and may be absent until near or after diagnosis (Schoettler and Williams, 2024). Severe TA-TMA can occur without overt schistocytosis (Wirtschafter et al., 2018). Accordingly, the 2022 standardized criteria no longer require schistocytosis as a mandatory diagnostic element and instead place greater emphasis on a composite assessment that includes markers more closely linked to the underlying pathophysiology of complement-mediated endothelial injury (Schoettler et al., 2023). This shift is pharmacologically important because it aligns disease assessment more closely with the mechanism than with a single morphologic feature.

In this context, soluble C5b-9 (sC5b-9) is particularly informative because it reflects terminal pathway activation and generation of the MAC, a key mediator of endothelial injury in TA-TMA. In adult allogeneic HSCT recipients, elevated serum sC5b-9 showed high sensitivity, supporting its value in serial assessment (Heybeli et al., 2020). However, because sC5b-9 is not universally available, additional biomarkers that capture upstream inflammatory activity are also of interest. Circulating double-stranded DNA (dsDNA), largely derived from neutrophil extracellular traps (NETs), is one such candidate (Arai et al., 2013). Elevated dsDNA early after transplantation has been associated with subsequent TA-TMA development, and persistent elevation later after HSCT has been linked to TA-TMA, GVHD, and mortality (Gloude et al., 2017). Given the close interplay between NETs formation, complement amplification, and endothelial injury, dsDNA may serve as a mechanistically relevant surrogate biomarker of disease activity.

Taken together, prospective serial assessment of hematologic markers of MAHA, platelet count, renal parameters, and, when available, complement-related biomarkers such as sC5b-9 or dsDNA provides an informed framework for identifying evolving TA-TMA activity and monitoring the effects of complement-directed therapeutic strategies.

## 4 Supportive and non–complement-directed therapies

As TA-TMA became increasingly recognized during the 2010s, various supportive and non–complement-directed therapies were attempted. Although case reports and small studies suggested benefit, their overall impact on prognosis remained limited, and most published reports were noncomparative.

### 4.1 Discontinuation or switching of CNIs

Using the 2022 harmonized criteria, discontinuation of CNIs or mTOR inhibitors in adult allogeneic HSCT recipients was associated with GVHD worsening in 37.5% of patients, and 86.7% of those with worsened GVHD died (Acosta-Medina et al., 2025). In a large retrospective study, overall survival (OS) was 3 months among patients who discontinued CNIs, versus 20 months among those switched to mycophenolate mofetil (MMF) (Heybeli et al., 2020). In multivariable analysis, switching CNIs to MMF independently contributed to TMA improvement, and in early TA-TMA, CNI-to-MMF switching was associated with lower mortality (Heybeli et al., 2020). In addition, a multicenter study suggested that continuation or careful reduction of CNIs was associated with better outcomes than switching from CNIs to corticosteroids, underscoring the need for caution when modifying CNIs (Matsui et al., 2020). Thus, simply discontinuing CNIs appears undesirable, potentially leading to GVHD-related death.

### 4.2 Plasma exchange

Plasma exchange (PE) was historically attempted in TA-TMA because of clinical overlap with immune-mediated TTP. However, TA-TMA typically lacks ADAMTS13 deficiency, limiting the rationale for PE. In a large cohort of 82 patients treated with PE, the overall response rate was 52%, but 100-day and 1-year OS were only 20% and 15%, respectively (Yang et al., 2022). In the 2023 American Society for Apheresis guidelines, PE for TA-TMA is graded as category III, grade 2C, reflecting low-quality evidence and an individualized rather than routine role (Connelly-Smith et al., 2023).

### 4.3 Defibrotide

Defibrotide is an endothelial-protective agent approved for sinusoidal obstruction syndrome/veno-occlusive disease (SOS/VOD) and has been used off-label for TA-TMA. In a single-center study, conventional management—predominantly defibrotide alone or combined with PE and/or rituximab—achieved an initial response rate of 61%, yet only 16% were long-term survivors, underscoring limited efficacy in advanced disease (Bohl et al., 2017). Defibrotide has shown responses in several reports and small studies, but increased bleeding complications have also been described (Martínez-Muñoz et al., 2019).

### 4.4 Rituximab

Rituximab, an anti-CD20 monoclonal antibody, is an established adjunctive therapy for immune-mediated TTP when combined with PE and immunosuppression. However, evidence in TA-TMA remains limited. In a retrospective series, 12 of 20 patients received rituximab, but its independent contribution could not be determined (Luo et al., 2023). Likewise, in a retrospective series including four TA-TMA cases treated with PE-based therapy with rituximab, mortality remained substantial (Gupta et al., 2024). Accordingly, rituximab may be considered case-by-case, but its efficacy has not been validated in prospective studies, and infectious risk must be weighed.

### 4.5 Recombinant thrombomodulin (rTM)

Recombinant thrombomodulin (rTM), approved in Japan for disseminated intravascular coagulation (DIC), has anticoagulant, anti-inflammatory, and endothelial-protective effects and has been explored as a non–complement-directed option for TA-TMA (Ito et al., 2019). A report suggested that rTM was associated with a median OS of approximately 4 months and improved OS (Fujiwara et al., 2016). However, evidence for rTM in established TA-TMA remains limited, and its efficacy has not been confirmed in prospective studies.

## 5 Complement-targeted therapies

Over the last decade, complement-targeted therapies aligned with TA-TMA pathophysiology have emerged and have been associated with improved outcomes compared with non–complement-directed therapies (Figure 1). In parallel, multiple complement-targeted agents have entered clinical development, and encouraging results from prospective and larger studies have begun to define their role in TA-TMA management (Table 1). However, direct cross-study comparisons should be interpreted with caution because published studies have enrolled heterogeneous populations with different severities, risk stratification, organ involvement, and response definitions.

**TABLE 1:** Summary of complement-targeted agents for transplant-associated thrombotic microangiopathy (TA-TMA).

Agent	Mechanism	Route	Study	Patient	Response	Survival outcomes
Eculizumab	C5 inhibition	IV	NCT03518203	Children and young adults	CR 48% PR 19%	6-month OS 71% 1-year OS 62%
Ravulizumab	Long-acting C5 inhibition	IV	NCT04557735 NCT04543591	Pediatric ≥12 years	met ≥1 response criterion 70.7% Ongoing	52-week OS 73.4% Ongoing
Narsoplimab	MASP-2 inhibition	IV	NCT02222545 NCT04247906	Adults Children & adults	ORR 61% Response: 67% in children; 69% in adults	100-day OS 68% 1-year OS 53.2% in children; 49.5% in adults
Nomacopan	C5 and LTB4 inhibition	SQ	NCT04784455	Pediatric	NR	NR
Iptacopan	Factor B inhibition	Oral	NCT07347990	≥12 years	Ongoing	Ongoing
Pegcetacoplan	C3 inhibition	SQ	NCT05148299	Adults	NR	NR

Abbreviations: MASP-2, mannose-binding lectin-associated serine protease-2; LTB4, leukotriene B4; IV, intravenous; SQ, subcutaneous; CR, complete response; PR, partial response; ORR, overall response rate; OS, overall survival; NR, not reported.

### 5.1 Eculizumab

Eculizumab is a humanized monoclonal antibody that inhibits terminal complement activation by binding C5, thereby preventing generation of C5a and formation of the MAC (Jodele and Sabulski, 2023). The first prospective multi-institutional eculizumab study in high-risk TA-TMA (NCT03518203) evaluated children and young adults with MODS and biomarker-defined high-risk disease (Jodele et al., 2024). With an intensified biomarker-guided dosing strategy for up to 24 weeks, survival was 71% at 6 months from diagnosis and 62% at 1-year post-transplant, with substantial organ recovery (Jodele et al., 2024). A large pediatric study also reported 1-year post-transplant survival of 66% in high-risk TA-TMA treated with eculizumab (Jodele et al., 2020). In adults, a retrospective study using the 2022 criteria reported an overall response rate (ORR) of 70% (complete response 50% and partial response 20%) with eculizumab (Acosta-Medina et al., 2025).

### 5.2 Ravulizumab

Ravulizumab is a long-acting, humanized anti-C5 monoclonal antibody engineered from eculizumab to provide sustained terminal complement inhibition. Its prolonged half-life enables extended maintenance dosing intervals (up to every 8 weeks). In a phase 3 trial (NCT04557735) involving pediatric patients with TA-TMA, ravulizumab achieved a 52-week OS of 73.4%. However, these findings should be interpreted with caution because enrollment did not require TA-TMA risk stratification, 68.3% of participants completed the 26-week treatment period, and 58.5% completed the 52-week study, and protocol-defined partial response was based on meeting at least 1 but not all TMA response criteria. At week 26, complete and partial TMA responses were observed in 17.1% and 53.7% of participants, respectively (Schoettler et al., 2026). A phase 3 trial (NCT04543591) enrolling adolescents and adults is ongoing.

### 5.3 Narsoplimab

Narsoplimab (OMS721) is a lectin pathway inhibitor targeting MASP-2 (mannose-binding lectin-associated serine protease-2). It received US approval in December 2025 as the first TA-TMA treatment. In a clinical trial enrolling adults with TA-TMA after allogeneic HSCT (NCT02222545), narsoplimab achieved an ORR of 61%, 100-day OS of 68%, and median OS of 274 days (Khaled et al., 2022). In a prospective expanded-access-program study (NCT04247906), estimated 1-year OS among pediatric allogeneic HSCT recipients was 53.2% (58.3% when narsoplimab was used first-line and 51.7% when used second-line or later) (Schoettler et al., 2025b). Among adult allogeneic HSCT recipients, estimated 1-year OS was 49.5% (52.7% in first-line and 42.7% in second-line or later) (Schoettler et al., 2025b). Because second-line narsoplimab cohorts included patients previously treated with eculizumab, these findings raise the possibility of activity after prior C5 inhibition (Schoettler et al., 2025b). However, this should be interpreted cautiously because assessment of eculizumab failure is not standardized, underexposure to eculizumab may occur in TA-TMA with aHUS-based dosing, management when switching to alternative complement therapies varies across practice settings, and validated biomarkers of lectin-pathway activation or effective MASP-2 blockade are not yet established.

### 5.4 Nomacopan

Nomacopan is a recombinant small protein that inhibits terminal complement activation by binding C5 and neutralizes leukotriene B4 (LTB4) (Sánchez-Tabernero et al., 2021). In TA-TMA, published evidence remains limited. A phase 3 open-label study of nomacopan in pediatric HSCT-TMA (NCT04784455) was initiated, but later terminated by the sponsor for non-medical reasons, and efficacy results have not been published.

### 5.5 Iptacopan

Iptacopan is an oral, first-in-class inhibitor of complement factor B that selectively blocks the alternative pathway by preventing factor B-dependent C3 and C5 convertase activity (Kavanagh et al., 2023). In complement-mediated disorders, iptacopan has shown efficacy in PNH and is being evaluated in the phase 3 study in aHUS (Kavanagh et al., 2023; Peffault de Latour et al., 2024). In TA-TMA, a phase 2 study (NCT07347990) is planned to evaluate iptacopan as second-line therapy, and prospective results are awaited.

### 5.6 Pegcetacoplan

Pegcetacoplan is a proximal complement inhibitor that binds complement component C3 and its activation fragment C3b, thereby inhibiting C3 cleavage and downstream complement activation (Hoy, 2021). In phase 3 trials in PNH, pegcetacoplan improved hemoglobin and transfusion-related outcomes and was superior to eculizumab in patients with persistent anemia despite prior C5 inhibition (Hillmen et al., 2021). In TA-TMA, a phase 2 study (NCT05148299) has been completed, although peer-reviewed efficacy results are not yet available.

## 6 Discussion

Complement-targeted therapy has reshaped the therapeutic landscape of TA-TMA by shifting management from empiric supportive care toward mechanism-based intervention. Accumulating data suggest that complement inhibition provides meaningful clinical benefit. Prospective multi-institutional data with eculizumab demonstrated improved survival and organ recovery, supporting terminal complement inhibition as an effective strategy. Likewise, narsoplimab improved laboratory markers and showed favorable survival, supporting upstream lectin pathway inhibition as another promising therapeutic approach.

Rapid expansion of complement therapeutics in other complement-mediated disorders suggests that the therapeutic horizon of TA-TMA may extend beyond the agents discussed here. Danicopan (factor D inhibitor) improved hemoglobin when added to C5 inhibitors in patients with PNH (Lee et al., 2023). Crovalimab (third-generation C5 inhibitor) demonstrated efficacy with subcutaneous administration in both complement inhibitor–naive and previously treated patients with PNH (Scheinberg et al., 2024; Röth et al., 2024). These findings indicate that alternative pathway and next-generation terminal complement inhibitors can provide clinical benefit across complement-driven diseases. Given the central role of complement dysregulation in TA-TMA, it is reasonable to hypothesize that some of these agents may also have activity. However, such extrapolation remains provisional. Differences in disease biology, transplant-related inflammatory triggers, organ involvement, and the need for rapid, sustained complement inhibition mean that efficacy in PNH or aHUS cannot be assumed to translate directly to TA-TMA. Dedicated prospective studies are therefore essential to define which agents, targets, and treatment settings are most appropriate for this complex transplant complication.

## 7 Conclusion

Complement-targeted therapies have substantially advanced TA-TMA treatment. Ongoing and future prospective studies will be essential to determine how individual complement inhibitors should be selected and integrated to further improve outcomes.

## References

[B1] Acosta-Medina A. A. Sridharan M. Go R. S. Moyer A. M. Leung N. Willrich M. A. V. (2025). Clinical outcomes and treatment strategies of adult transplant-associated thrombotic microangiopathy: external validation of harmonizing definitions and high-risk criteria. Am. J. Hematol. 100, 830–839. 10.1002/ajh.27651 40047384 PMC11966343

[B2] Arai Y. Yamashita K. Mizugishi K. Watanabe T. Sakamoto S. Kitano T. (2013). Serum neutrophil extracellular trap levels predict thrombotic microangiopathy after allogeneic stem cell transplantation. Biol. Blood Marrow Transpl. 19, 1683–1689. 10.1016/j.bbmt.2013.09.005 24055655

[B3] Bohl S. R. Kuchenbauer F. von Harsdorf S. Kloevekorn N. Schönsteiner S. S. Rouhi A. (2017). Thrombotic microangiopathy after allogeneic stem cell transplantation: a comparison of eculizumab therapy and conventional therapy. Biol. Blood Marrow Transpl. 23, 2172–2177. 10.1016/j.bbmt.2017.08.019 28860002

[B4] Connelly-Smith L. Alquist C. R. Aqui N. A. Hofmann J. C. Klingel R. Onwuemene O. A. (2023). Guidelines on the use of therapeutic apheresis in clinical practice - evidence-based approach from the Writing Committee of the American Society for Apheresis: the ninth special issue. J. Clin. Apher. 38, 77–278. 10.1002/jca.22043 37017433

[B5] Dandoy C. E. Rotz S. Alonso P. B. Klunk A. Desmond C. Huber J. (2021). A pragmatic multi-institutional approach to understanding transplant-associated thrombotic microangiopathy after stem cell transplant. Blood Adv. 5, 1–11. 10.1182/bloodadvances.2020003455 33570619 PMC7805323

[B6] Dvorak C. C. Higham C. Shimano K. A. (2019). Transplant-associated thrombotic microangiopathy in pediatric hematopoietic cell transplant recipients: a practical approach to diagnosis and management. Front. Pediatr. 7, 133. 10.3389/fped.2019.00133 31024873 PMC6465621

[B8] Epperla N. Li A. Logan B. Fretham C. Chhabra S. Aljurf M. (2020). Incidence, risk factors for and outcomes of transplant-associated thrombotic Microangiopathy. Br. J. Haematol. 189, 1171–1181. 10.1111/bjh.16457 32124435 PMC7726817

[B9] Fujiwara H. Maeda Y. Sando Y. Nakamura M. Tani K. Ishikawa T. (2016). Treatment of thrombotic microangiopathy after hematopoietic stem cell transplantation with recombinant human soluble thrombomodulin. Transfusion 56, 886–892. 10.1111/trf.13437 26711692

[B10] Gloude N. J. Khandelwal P. Luebbering N. Lounder D. T. Jodele S. Alder M. N. (2017). Circulating dsDNA, endothelial injury, and complement activation in thrombotic microangiopathy and GVHD. Blood 130, 1259–1266. 10.1182/blood-2017-05-782870 28705839 PMC5714230

[B11] Gupta D. Moule P. Ranjan V. Kotwal J. Khillan K. Saraf A. (2024). Clinical profile, treatment and outcome of Thrombotic Thrombocytopenia Purpura (TTP) in Rituximab Era-an experience from tertiary care Centre from North India. Indian J. Hematol. Blood Transfus. 40, 655–659. 10.1007/s12288-024-01775-1 39469158 PMC11512964

[B12] Heybeli C. Sridharan M. Alkhateeb H. B. Villasboas Bisneto J. C. Buadi F. K. Chen D. (2020). Characteristics of late transplant-associated thrombotic microangiopathy in patients who underwent allogeneic hematopoietic stem cell transplantation. Am. J. Hematol. 95, 1170–1179. 10.1002/ajh.25922 32618000

[B13] Higham C. S. Collins G. Shimano K. A. Melton A. Kharbanda S. Winestone L. E. (2021). Transplant-associated thrombotic microangiopathy in pediatric patients: pre-HSCT risk stratification and prophylaxis. Blood Adv. 5, 2106–2114. 10.1182/bloodadvances.2020003988 33877298 PMC8095147

[B14] Hillmen P. Szer J. Weitz I. Röth A. Höchsmann B. Panse J. (2021). Pegcetacoplan versus eculizumab in paroxysmal nocturnal hemoglobinuria. N. Engl. J. Med. 384, 1028–1037. 10.1056/NEJMoa2029073 33730455

[B15] Ho V. T. Cutler C. Carter S. Martin P. Adams R. Horowitz M. (2005). Blood and marrow transplant clinical trials network toxicity committee consensus summary: thrombotic microangiopathy after hematopoietic stem cell transplantation. Blood Marrow Transpl. 11, 571–575. 10.1016/j.bbmt.2005.06.001 16041306

[B16] Hoy S. M. (2021). Pegcetacoplan: first approval. Drugs 81, 1423–1430. 10.1007/s40265-021-01560-8 34342834

[B17] IbrahimovaA. LuebberingN. StreckerL. LangenbergL. AbdullahS. LakeK. E. (2026). Neutrophil extracellular traps, endothelial injury and use of abatacept for graft versus host disease prophylaxis. Haematologica. 10.3324/haematol.2024.287235 41709743

[B18] Ito T. Thachil J. Asakura H. Levy J. H. Iba T. (2019). Thrombomodulin in disseminated intravascular coagulation and other critical conditions-a multi-faceted anticoagulant protein with therapeutic potential. Crit. Care 23, 280. 10.1186/s13054-019-2552-0 31416465 PMC6694689

[B19] Jodele S. Sabulski A. (2023). Reeling in complement in transplant-associated thrombotic microangiopathy: you're going to need a bigger boat. Am. J. Hematol. 98 (Suppl. 4), S57–S73. 10.1002/ajh.26872 36746623

[B21] Jodele S. Zhang K. Zou F. Laskin B. Dandoy C. E. Myers K. C. (2016). The genetic fingerprint of susceptibility for transplant-associated thrombotic microangiopathy. Blood 127, 989–996. 10.1182/blood-2015-08-663435 26603840 PMC4828073

[B22] Jodele S. Dandoy C. E. Lane A. Laskin B. L. Teusink-Cross A. Myers K. C. (2020). Complement blockade for TA-TMA: lessons learned from a large pediatric cohort treated with eculizumab. Blood 135, 1049–1057. 10.1182/blood.2019004218 31932840 PMC7099329

[B23] Jodele S. Aguayo-Hiraldo P. Dandoy C. E. Lane A. Teusink-Cross A. Sabulski A. (2024). A prospective multi-institutional study of eculizumab to treat high-risk stem cell transplantation-associated TMA. Blood 143, 1112–1123. 10.1182/blood.2023022526 37946262 PMC10972707

[B24] Kafa K. Hoell J. I. (2024). Transplant-associated thrombotic microangiopathy in pediatrics: incidence, risk factors, therapeutic options, and outcome based on data from a single center. Front. Oncol. 14, 1399696. 10.3389/fonc.2024.1399696 39050576 PMC11266128

[B25] Kavanagh D. Greenbaum L. A. Bagga A. Karki R. G. Chen C. W. Vasudevan S. (2023). Design and rationale of the APPELHUS phase 3 open-label study of factor B inhibitor iptacopan for atypical hemolytic uremic syndrome. Kidney Int. Rep. 8, 1332–1341. 10.1016/j.ekir.2023.04.029 37441479 PMC10334406

[B26] Khaled S. K. Claes K. Goh Y. T. Kwong Y. L. Leung N. Mendrek W. (2022). Narsoplimab, a mannan-binding lectin-associated serine protease-2 inhibitor, for the treatment of adult hematopoietic stem-cell transplantation-associated thrombotic microangiopathy. J. Clin. Oncol. 40, 2447–2457. 10.1200/JCO.21.02389 35439028 PMC9467678

[B27] Laskin B. L. Denburg M. R. Furth S. L. Moatz T. Altrich M. Kleiboeker S. (2020). The natural history of BK polyomavirus and the host immune response after stem cell transplantation. Clin. Infect. Dis. 71, 3044–3054. 10.1093/cid/ciz1194 31851312 PMC7819507

[B28] Lee J. W. Griffin M. Kim J. S. Lee Lee L. W. Piatek C. Nishimura J. I. (2023). Addition of danicopan to ravulizumab or eculizumab in patients with paroxysmal nocturnal haemoglobinuria and clinically significant extravascular haemolysis (ALPHA): a double-blind, randomised, phase 3 trial. Lancet Haematol. 10, e955–e965. 10.1016/S2352-3026(23)00315-0 38030318

[B29] Liu W. Zhu X. Xiao Y. (2024). Neurological involvement in hematopoietic stem cell transplantation-associated thrombotic microangiopathy. Ann. Hematol. 103, 3303–3313. 10.1007/s00277-024-05798-6 38763940 PMC11358180

[B30] Luo L. Xiong H. Chen Z. Yang L. Sun M. Lu W. (2023). Clinical characteristics of pediatric allogeneic hematopoietic stem cell transplantation-associated thrombotic microangiopathy (TA-TMA): a retrospective single-center analysis. Clin. Transl. Oncol. 25, 2451–2461. 10.1007/s12094-023-03129-1 36973479 PMC10293339

[B31] Mahmoudjafari Z. Alencar M. C. Alexander M. D. Johnson D. J. Yeh J. Evans M. D. (2023). Hematopoietic stem cell transplantation-associated thrombotic microangiopathy and the role of advanced practice providers and pharmacists. Bone Marrow Transpl. 58, 625–634. 10.1038/s41409-023-01951-3 37059738 PMC10247375

[B32] Martínez-Muñoz M. E. Forés R. Lario A. Bautista G. Bueno J. L. de Miguel C. (2019). Use of defibrotide to treat adult patients with transplant-associated thrombotic microangiopathy. Bone Marrow Transpl. 54, 142–145. 10.1038/s41409-018-0256-8 29899573

[B33] Matsui H. Arai Y. Imoto H. Mitsuyoshi T. Tamura N. Kondo T. (2020). Risk factors and appropriate therapeutic strategies for thrombotic microangiopathy after allogeneic HSCT. Blood Adv. 4, 3169–3179. 10.1182/bloodadvances.2020001703 32658984 PMC7362379

[B35] Peffault de Latour R. Röth A. Kulasekararaj A. G. Han B. Scheinberg P. Maciejewski J. P. (2024). Oral iptacopan monotherapy in paroxysmal nocturnal hemoglobinuria. N. Engl. J. Med. 390, 994–1008. 10.1056/NEJMoa2308695 38477987

[B36] Röth A. He G. Tong H. Lin Z. Wang X. Chai-Adisaksopha C. (2024). Phase 3 randomized COMMODORE 2 trial: crovalimab versus eculizumab in patients with paroxysmal nocturnal hemoglobinuria naive to complement inhibition. Am. J. Hematol. 99, 1768–1777. 10.1002/ajh.27412 38884175

[B37] Ruutu T. Barosi G. Benjamin R. J. Clark R. E. George J. N. Gratwohl A. (2007). Diagnostic criteria for hematopoietic stem cell transplant-associated microangiopathy: results of a consensus process by an International Working Group. Haematologica 92, 95–100. 10.3324/haematol.10699 17229640

[B38] Sánchez-Tabernero S. Fajardo-Sanchez J. Weston-Davies W. Parekh M. Kriman J. Kaye S. (2021). Dual inhibition of complement component 5 and leukotriene B4 by topical rVA576 in atopic keratoconjunctivis: TRACKER phase 1 clinical trial results. Orphanet J. Rare Dis. 16, 270. 10.1186/s13023-021-01890-6 34116700 PMC8196439

[B39] Scheinberg P. Clé D. V. Kim J. S. Nur E. Yenerel M. N. Barcellini W. (2024). Phase 3 randomized COMMODORE 1 trial: crovalimab versus eculizumab in complement inhibitor-experienced patients with paroxysmal nocturnal hemoglobinuria. Am. J. Hematol. 99, 1757–1767. 10.1002/ajh.27413 38924124

[B40] Schoettler M. L. Williams K. M. (2024). Application of the consensus definition of transplant associated thrombotic Microangiopathy (TA-TMA) requires a more expansive lens to accurately diagnose and risk stratify Patients—Beware reliance on schistocytes alone. Transpl. Cell. Ther. 30, 818–820. 10.1016/j.jtct.2024.05.027 38838782

[B41] Schoettler M. L. Carreras E. Cho B. Dandoy C. E. Ho V. T. Jodele S. (2023). Harmonizing definitions for diagnostic criteria and prognostic assessment of transplantation-associated thrombotic microangiopathy: a report on behalf of the European Society for Blood and Marrow Transplantation, American Society for Transplantation and Cellular Therapy, Asia-Pacific Blood and Marrow Transplantation Group, and Center for International Blood and Marrow Transplant Research. Transpl. Cell Ther. 29, 151–163. 10.1016/j.jtct.2022.11.015 PMC10119629 36442770

[B42] Schoettler M. L. Gavriilaki E. Carreras E. Cho B. K. Dandoy C. E. Ho V. T. (2025a). An ASTCT, CIBMTR, EBMT, and APBMT consensus statement defining response criteria for hematopoietic cell transplantation associated thrombotic microangiopathy (TA-TMA) directed therapy. Transpl. Cell Ther. 31, 610–623. 10.1016/j.jtct.2025.05.028 40514012 PMC12718118

[B43] Schoettler M. L. Pusarla S. K. Nangia N. Perales M. A. Duarte R. F. Grauer A. (2025b). Narsoplimab results in excellent survival in adults and children with hematopoietic cell transplant associated thrombotic microangiopathy (TA-TMA). Am. J. Hematol. 100, 2040–2051. 10.1002/ajh.70044 40880162 PMC12516664

[B44] Schoettler M. L. Dvorak C. C. Bueno-Sánchez D. Krauss A. Chaudhury S. Bachman E. (2026). Ravulizumab plus best supportive care in pediatric patients with hematopoietic stem cell transplantation-associated thrombotic microangiopathy: 52-week results from a phase 3 trial. Transpl. Cell Ther. 32, S69–S70. 10.1016/j.jtct.2025.12.104

[B45] Van Benschoten V. Roy C. Gupta R. Ouellette L. Hingorani S. Li A. (2022). Incidence and risk factors of transplantation-associated thrombotic microangiopathy: a systematic review and meta-analysis. Transpl. Cell Ther. 28, 266.e1–266.e8. 10.1016/j.jtct.2022.01.009 35042011

[B47] Vasu S. Zhao Q. Miller E. G. Elder P. Langenberg L. Cataland S. (2025). High incidence of severe TA-TMA increases mortality in adult allogeneic transplant recipients: a prospective MIDAS Consortium study. Blood 146, 638–646. 10.1182/blood.2025028390 40300075

[B48] Wirtschafter E. VanBeek C. Linhares Y. (2018). Bone marrow transplant-associated thrombotic microangiopathy without peripheral blood schistocytes: a case report and review of the literature. Exp. Hematol. Oncol. 7, 14. 10.1186/s40164-018-0106-9 29977661 PMC6013965

[B49] Yang L. P. Zhao P. Wu Y. J. Fu H. X. He Y. Mo X. D. (2022). Treatment outcome and efficacy of therapeutic plasma exchange for transplant-associated thrombotic microangiopathy in a large real-world cohort study. Bone Marrow Transpl. 57, 554–561. 10.1038/s41409-022-01581-1 35079139

[B50] Yuen J. Pluthero F. G. Douda D. N. Riedl M. Cherry A. Ulanova M. (2016). NETosing neutrophils activate complement both on their own NETs and bacteria via alternative and non-alternative pathways. Front. Immunol. 7, 137. 10.3389/fimmu.2016.00137 27148258 PMC4831636

